# Factors associated with breeding site fidelity in the sex-role reversed red phalarope

**DOI:** 10.1093/beheco/arag090

**Published:** 2026-07-28

**Authors:** Peter Santema, Johannes Krietsch, Bart Kempenaers

**Affiliations:** Department of Ornithology, Max Planck Institute for Biological Intelligence, Eberhard-Gwinner Strasse, 82319, Seewiesen, Germany; Department of Ornithology, Max Planck Institute for Biological Intelligence, Eberhard-Gwinner Strasse, 82319, Seewiesen, Germany; Department of Coastal Systems, NIOZ Royal Netherlands Institute for Sea Research, PO Box 59, 1790 AB, Den Burg, Netherlands; Department of Ornithology, Max Planck Institute for Biological Intelligence, Eberhard-Gwinner Strasse, 82319, Seewiesen, Germany

**Keywords:** arctic, breeding dispersal, polyandry, red phalarope, sex-role reversal, site fidelity

## Abstract

When birds breed in multiple years, they can either return to their former breeding site or disperse to a new area. Consistent with theoretical predictions, the degree of breeding site fidelity often differs between the sexes in relation to the mating system of a species. However, most research has focused on species with conventional sex roles. Studying socially polyandrous species, in which typical sex roles are reversed, may provide insights into the drivers of breeding site fidelity in each sex. We color-banded 169 male and 184 female red phalaropes *Phalaropus fulicarius*, a species in which females compete for access to males (*female access polyandry*), and examined factors that predicted their return to the study area. Breeding site fidelity was relatively low, but substantially higher in males (20.7%) than in females (4.3%). For both sexes, the probability of returning was associated with local breeding performance: males that attended a nest in the study area were more likely to return than males without a nest, and females that locally bred with 2 males were more likely to return than those that only produced a clutch for a single male. Our findings are consistent with the prediction that site fidelity should be male-biased in species with a female-access polyandrous mating system. Our study also highlights that breeding site fidelity is associated with local breeding success, even in a species that shows a reversal of typical sex-roles, where individuals do not defend a territory, and breed in an environment that is highly unpredictable between years.

## Teaser

We show that, in the sex-role reversed red phalarope, which shows male-only parental care, breeding site fidelity was substantially higher in males than in females. For both sexes, breeding site fidelity was associated with local breeding performance. Our findings are thus consistent with the prediction that breeding site fidelity should be male-biased in species where females compete for access to males.

## Introduction

When birds breed in multiple years, they can either return to their previous breeding location or disperse to a new one (Greenwood 1980). The level of breeding site fidelity can have important consequences for evolutionary dynamics and population processes (Clobert et al. 2001; Bowler and Benton 2005). For example, extensive breeding dispersal promotes gene flow between breeding areas, which reduces the potential for local adaptation and the evolution of subspecies (Hanski 1999; D'Urban Jackson et al. 2017; Kempenaers and Valcu 2017). By increasing contact between individuals from different geographic regions, breeding dispersal also facilitates the transmission of infectious diseases (Clobert et al. 2001; Owen et al. 2021; Jeglinski et al. 2024). Additionally, breeding dispersal influences a species’ capacity for range expansion and the establishment of new breeding sites (Fahrig and Merriam 1994; Merkle et al. 2022).

For an individual, being faithful to a particular breeding site, rather than dispersing, can have several potential benefits. For instance, site fidelity increases familiarity with local conditions, which may enhance foraging efficiency, facilitate efficient use of resources, and improve predator avoidance (Vlasak 2006; Brown et al. 2008; Olsson and Brown 2010; Piper 2011). In territorial species, returning to the same breeding ground may also confer dominance over conspecific competitors due to a “residency advantage” (Switzer 2004; Kokko et al. 2006). Furthermore, returning to the same breeding site facilitates re-uniting with the previous breeding partner, which may increase reproductive success through breeding earlier in the season or as a result of better coordination of territory defense or parental care (Adkins-Regan and Tomaszycki 2007; Griggio et al. 2011; Sánchez-Macouzet et al. 2014; Griffith 2019). However, there are also potential costs to being site faithful. Being faithful to a particular area may prevent an individual from taking advantage of better conditions elsewhere (Switzer 1993). Moreover, it may increase the chance of mating or competing for resources with genetic relatives (Hamilton and May 1977; Perrin and Mazalov 1999).

The degree of breeding site fidelity differs markedly between species, populations, and individuals (Greenwood 1980; Greenwood and Harvey 1982; Paradis et al. 1998). Although the factors underlying variation in breeding site fidelity remain debated and likely involve multiple aspects of the ecology and life-history of a species (Dobson 2013; Trochet et al. 2016; Li and Kokko, 2019), site fidelity of each sex is predicted to vary with the mating system (Pitelka et al. 1974; Greenwood 1980). In species with a resource defense mating system, individuals of the competitive sex (usually males) are expected to show higher site fidelity because they benefit more from site familiarity or from a prior residency advantage over competitors (Greenwood 1980; Clarke et al. 1997). In contrast, in species with a mate-defense mating system, individuals of the competitive sex are expected to show lower site fidelity, because they do not need to defend a resource and benefit from seeking mates wherever they are available (Greenwood 1980; Clarke et al. 1997). Another relevant aspect of the mating system is the division of parental responsibilities; individuals of the sex that invests more in parental care are expected to benefit more from local familiarity, thus favoring site fidelity (Greenwood 1980; Trochet et al. 2016). Whether or not an individual returns to a particular breeding site may also vary with the reproductive success it experienced at that site, as breeding success may be an important indicator of the quality of a site or mate (Gavin and Bollinger 1988; Clobert et al. 2001).

Empirical findings are generally consistent with these predictions (Greenwood and Harvey 1982; Clarke et al. 1997; Haas 1998; Hoover 2003). However, studies on site fidelity in birds have been conducted primarily on socially monogamous species with biparental care, which comprise the majority of species (>90%, Lack 1968; Reynolds et al. 2002). Factors underlying breeding site fidelity may be different in species with a different mating system. For instance, in polygamous species in which only 1 parent provides parental care, the benefits of mate reunion are unlikely to play a role. Accordingly, breeding site fidelity is typically much lower in polygamous species than in monogamous species, at least among shorebirds (Kwon et al. 2022). A few studies examined site fidelity in polygynous species with female-only care, in which males defend a territory or position on a lek to which they try to attract females. These studies reported that breeding site fidelity was higher in males and correlated positively with breeding success, consistent with predictions (Lill 1974; Santema et al. 2025). Nevertheless, studying species with a different mating system may be informative for understanding the factors promoting breeding site fidelity.

In socially polyandrous species, the typical sex-roles are reversed: females compete for access to males and males provide all parental care (Oring 1986). Predictions regarding the sex which shows higher site fidelity are thus also reversed. In species in which females defend a territory to which they try to attract males (*resource defense polyandry*), females are expected to show higher site fidelity. In contrast, in species in which females defend individual mates rather than resources (*female access polyandry*), males should show higher site fidelity (Greenwood 1980). However, only few studies have examined breeding site fidelity in such species, possibly because social polyandry is rare, occurring in fewer than 1% of bird species (Reynolds et al. 2002). Two studies on the red-necked phalarope *Phalaropus lobatus*, a species with a female access polyandrous mating system, found that return rates did not differ significantly between males and females (Reynold and Crooke 1988; Schamel and Tracy 1991). One of these studies found that males—but not females—were more likely to return after a successful breeding attempt (Reynold and Crooke 1988), but site fidelity was unrelated to breeding success in either sex in the other study (Schamel and Tracy 1991). In the closely related red phalarope (*Phalaropus fulicarius*), which has a similar mating system, local return rates were somewhat higher in males than in females but this difference was not statistically significant, and the limited sample sizes precluded further examination of factors associated within breeding site fidelity (Schamel and Tracy 1991). Thus, overall, little is known about factors associated with breeding site fidelity in polyandrous species.

Here, we report on a study of breeding site fidelity in the red phalarope. Over a period of 2 breeding seasons, we caught 169 males and 184 females at a breeding site in the Arctic and monitored their presence in the same area in the following seasons. We also monitored all nesting attempts and genotyped all adults and offspring, such that we could unambiguously assign each nesting attempt to individual females. First, we tested whether male and female red phalaropes differed in their likelihood to return. Second, we examined whether local breeding success and measures of individual quality were associated with the likelihood of return to the same breeding area in following years. Third, we examined whether individuals that had returned (ie been at the study site in a previous season) differed in breeding success and measures of individual quality from those that had not been recorded in the same breeding area previously. If both members of a pair returned, we determined whether they bred together again.

## Materials and methods

### Study species

The red phalarope is a pelagic wader that lives in (sub-)tropical marine habitats most of the year, but migrates to the Arctic tundra in summer for breeding (Tracy et al. 2020; Saalfeld et al. 2024). Because of their northerly breeding range, red phalaropes experience considerable interannual variation in local breeding conditions (eg food resources, predation risk), and late snowmelt may locally preclude nesting altogether in some years (Mayfield 1978; Schamel and Tracy 1987, 1991). The typical sex roles are reversed in red phalaropes: females compete more strongly for mating opportunities and are larger and more brightly colored than males, and males provide all parental care (Tracy et al. 2020; Delhey et al. 2024). Red phalaropes do not defend a territory, but both pair members defend their partner against approaching conspecifics, involving fights with both same- and opposite-sex individuals (Tracy et al. 2020). Individuals form short-term pair bonds that are characterized by close proximity, frequent communication with contact calls, and frequent copulation (Schamel and Tracy 1977; Krietsch et al. 2024). Females typically leave their mate immediately after finishing their clutch and—if unpaired males are available—mate with a different male. Females can thus produce multiple clutches (up to 3) per season, whereas males can only raise 1 brood successfully (Schamel and Tracy 1977; Krietsch et al. 2022).

Previous work examining breeding site fidelity in red phalaropes reported low site fidelity (Schamel and Tracy 1991): at the same study site as ours (see below), local return rates were 9% for males (6 out of 66) and 3% for females (1 out of 30), while at a different site (Cape Espenberg, Alaska) local return rates were 16% (6 out of 38 males) and 10% (2 out of 20 females). A study in an area near our study site reported a male return rate of 12% (28 out of 240, Saalfeld and Lanctot 2015), while a study across 4 sites in Alaska and Canada reported a return rate of 10% (27 out of 269 males, Weiser et al. 2018). Finally, a study that color-banded 4 males at a small, isolated population in north-east Iceland reported that all 4 individuals returned to the same breeding area between years (Whitfield 1995). While the findings of Schamel and Tracy (1991) suggest that males may have higher return rates than females, the difference they report was not statistically significant, and it thus remains unclear whether breeding site fidelity in red phalaropes is sex-biased. Moreover, there is no information on factors associated with breeding site fidelity in either males or females of this species (Tracy et al. 2020).

### General procedures

We studied red phalaropes in a 2.5 km^2^ plot of open, wet tundra habitat near Utqiagvik (formerly Barrow), Alaska (71°19′N, 156°39′W), during the breeding seasons of 2017 to 2019. Each season, we attempted to catch all males and females soon after they arrived at the study site using handheld mistnets. To minimize potential disruption of breeding, all birds were caught away from their nest. Upon capture, we banded each individual with a metal band and a unique combination of 4 color bands, and measured its body mass (to the nearest 0.1 g), tarsus length, culmen length (both to the nearest 0.1 mm), and wing length (to the nearest 0.5 mm). We also took a 5 to 10 μl blood sample by puncturing the brachial vein with a sterile needle and using a small capillary tube. We stored the blood in 1 ml Queen's lysis buffer (Seutin et al. 1991) for later genetic sexing and parentage analysis (see Krietsch et al. 2022). We also collected 50 to 200 μl of blood in heparinized microhematocrit capillary tubes. On the day of collection, after returning from the field, we centrifuged these samples at 5,000 rpm for 10 min to separate plasma from red blood cells. We then measured hematocrit levels as the percentage of packed red blood cells over the total sample (see Kempenaers and Valcu 2017 for details). For females, we also recorded when an egg was detected in the abdomen by palpation. When an individual was captured more than once within a breeding season (7.3% of females and 7.4% of males), the measurements taken at the first capture were used for analyses. As part of an investigation into red phalarope movements, 22 females (18 in 2017 and 4 in 2018) included in this study were equipped with 2 g solar-powered Argos platform transmitter terminal (PTT) tags (Microwave Telemetry, Inc.). In addition, 48 females and 51 males were equipped with 3.5 g solar-powered NanoRadioTag-3 telemetry loggers (Milsar Inc.) in 2018. PTT tags were attached using a wing-loop harness made of 1.9 mm diameter Teflon held together with 3 crimps (2017) or medical-grade silicone tubing (2018), while Milsar tags were mounted on a piece of goatskin and glued to the bird's back after trimming some body feathers, using a flexible nontoxic glue.

Every day throughout each breeding season, up to 10 observers resighted color-banded individuals by systematically walking through the entire study area, with at least 8 observers in the field daily between 4 and 27 June in every year (see Table S1 for details). Each day, we also searched for nests in the study area by (i) following a male or a pair and observing them make or sit in a nest scrape, (ii) following a female until she went to a nest to lay an egg, (iii) following a male after flushing him off a nest or during a natural incubation break until he returned to the nest, and (iv) inspecting potential nest locations based on location data of males tagged with a GPS transmitter (Krietsch et al. 2024). Most nests were found soon after nest initiation (78% before clutch completion; Krietsch et al. 2022), and no new nests were found by systemically rope-dragging the study area near the end of the breeding season, suggesting that nest detection was (near-)complete. Moreover, most newly discovered nests (87%) belonged to already banded males, and at most nests (93%) the female had also been caught, indicating that most adults present in the study area were captured early in the season (Krietsch et al. 2022). Given the high risk of nest predation in our study area, we collected clutches after completion and replaced them with dummy eggs, to ensure that we obtained DNA samples from all offspring. We placed the collected eggs in an incubator (Grumbach Compact SA) and moved them to a hatcher when eggs showed signs of pipping. Soon after hatching, when the chicks were dry, we banded them with a uniquely numbered metal band and took a small (3 to 5 μl) blood sample from the jugular vein using a 0.3 × 13 mm sterile needle on a syringe. We also stored these blood samples in Queen's lysis buffer. Within a few hours after hatching, we returned chicks to the nest and removed the dummy eggs; males always accepted the chicks and immediately started brooding them. We opened eggs that did not hatch, collected a tissue sample from the dead embryos and stored it in RNAlater (Sigma-Aldrich). We determined parentage of chicks and unhatched eggs using a set of 30 highly variable microsatellite markers, which allowed us to unequivocally assign nests to individual females (for details, see Krietsch et al. 2022). We cannot exclude the possibility that the artificial incubation procedure may have influenced nesting success and hence the local return rates of males. Depredation of dummy clutches was common (30% of nests), but predation of real clutches could even have been higher.

All procedures were approved by the US Geological Survey Bird Banding Laboratory (permit number 23520), the Alaska Department of Fish and Game (permit numbers 17-149, 18-146, and 19-143), the US Fish and Wildlife Service (permit number MB210494-0), and the North Slope Borough and Ukpeaġvik Iñupiat Corporation.

### Data processing

We estimated an individual's tenure at the study site as the number of days between the first observation (arrival) and the last observation (departure) of that individual. For most birds, we considered the day it was first captured as its arrival date. However, if an individual was associated with a previously found nest when it was first captured (*N* = 20 males, 16 females), we used the date of nest initiation as its arrival date (see Krietsch et al. 2022). If the nest an individual was associated with was still active after the individual was last observed (*N* = 39 males, 52 females), we used the date of clutch completion (for females) or the last date on which the nest was known to be active (for males) as that individual's departure date.

A nest was considered to have successfully produced hatchlings if it was tended by the male throughout the incubation period until we replaced the dummy eggs with chicks that had hatched in the incubator. We use this as our definition of nest success when examining associations between breeding success and site fidelity in males. We thus assume that the male perceived the presence of chicks in the nest as successful hatching of its clutch, which is supported by the fact that all males accepted the chicks and cared for them.

The majority of individuals that returned to the study area did so in the next year, but 4 males and 1 female that were caught in 2017 were observed on the study site in 2019 but not in 2018. Because birds that returned in nonconsecutive years still can be considered showing site-fidelity, we included all individuals that returned (after either 1 or 2 yr) as site-faithful in the analyses. The results are similar and the conclusions remain the same when only individuals that returned the following year were considered site-faithful (details not shown).

For individuals that returned to the study site and were associated with a nest in both years, we defined dispersal distance as the distance (in meters) between their nest locations. Estimating dispersal distances is valuable because little is known about (sex differences in) dispersal in polyandrous species. Moreover, it is methodologically relevant because the dispersal distance is related to the likelihood that a returning individual breeds again within the study plot and, thus, to our estimates of return rates. For individuals that had more than 1 nest in the initial year (*N* = 1 male, 1 female) or in the year of their return (*N* = 4 males, 1 female), we used the last nest of the initial year and the first nest in the year of their return to calculate dispersal distance.

An egg was detected in the abdomen by palpation in 13% of all caught females. Because each egg represents ∼13% of a female's body mass, female body mass may fluctuate substantially depending on whether or not an egg is present. For analyses that included body condition as either an explanatory or a response variable, we therefore ran the models (i) including all females and (ii) excluding females carrying an egg. This exclusion did not affect any of the results (details not shown) and we therefore only report the results of the models that included all females.

### Statistical analyses

All statistical analyses were performed with R (version 4.2.2, R Core Team 2024, www.r-project.org). We performed (generalized) linear models using the lme4 package (Bates et al. 2015). For binomial models, coefficients reported in the tables and text are on the logit (link) scale, while predicted values shown in figures are transformed to the response scale (ie probabilities). To prepare figures, we used the ggplot2 package (Wickham 2016).

To evaluate whether males and females differed in their likelihood of returning to the study area, we ran a generalized linear model with a binomial error distribution, with whether or not an individual returned as the response variable and with the sex of the individual as the explanatory variable. We included year (a factor with 2 levels) as a fixed effect, along with a sex × year interaction, to examine whether return rates differed between the 2 yr in a sex-specific fashion. Year was treated as a fixed effect, rather than a random effect, because the small number of years (*n* = 2) precludes reliable estimation of a random-effect variance, and modeling year as fixed effect allows to test the sex × year interaction. To evaluate whether males and females differed in their dispersal distance, we ran a linear model with dispersal distance as the response variable and with the sex of the individual as the explanatory variable.

To examine whether local breeding success and measures of individual quality were associated with the likelihood of return, we ran a generalized linear model with a binomial error distribution, with returning (yes/no) as the response variable and with the following explanatory variables measured in the year of first observation (year X): (i) nesting in the study area (yes/no), (ii) body size, (iii) body condition, (iv) hematocrit level, and (v) whether or not a bird was tagged. As a measure for body size, we used the first principal component of tarsus length, wing length, and culmen length. As a measure for body condition, we used the residuals from a linear regression of body mass on body size. Both body size and body condition were calculated separately for each sex. As the factors underlying breeding site fidelity may differ between the sexes, we ran this model separately for males and females.

For males that nested in the study area, we then examined whether the likelihood of returning was associated with whether or not they had bred successfully. We ran a generalized linear model with returning (yes/no) as the response variable, and with nesting success in year X (male incubating until receiving chicks: yes/no) as the explanatory variable. For females that nested in the study area, we examined whether the likelihood that they returned was associated with whether they were socially polyandrous, ie produced a clutch for more than 1 male in the study area. We ran a generalized linear model with returning (yes/no) as the response variable, and with polyandry in year X (yes/no) as the explanatory variable. Females that had laid eggs for only 1 male in the study area were considered socially monogamous, although it is possible that they previously or subsequently had mated with a male outside the study area. Mating status (monogamous or polyandrous) thus only refers to the local situation. Body size, body condition, hematocrit level, and whether or not a bird was tagged were also included as explanatory variables in both models.

To test whether individuals that had been present in the study area in a previous year differed in the probability of nesting in the study area, in arrival date, and in body condition in year X + 1 or X + 2 from individuals that were observed for the first time, we ran (generalized) linear models with the variable of interest as the response variable and with presence in a previous year (yes/no) as the explanatory variable. To account for potential seasonal changes in body condition, we included capture date (centered within each season) as an additional explanatory variable in the model examining body condition. Because the consequences of breeding site fidelity may differ between the sexes, we ran these models separately for males and females. For males that nested in the study area, we also ran a model to test whether those that had been present in the study area in a previous year differed in the probability of breeding successfully (incubating until receiving hatchlings) in the year of return compared with those who had not been observed in previous years. For females that nested in the study area, we ran a model to test whether those that had been present in the study area in a previous year differed in the probability of being socially polyandrous in the year of return compared with those that had not been observed in previous years.

## Results

### Sex difference in local return rates

**Figure 1:**
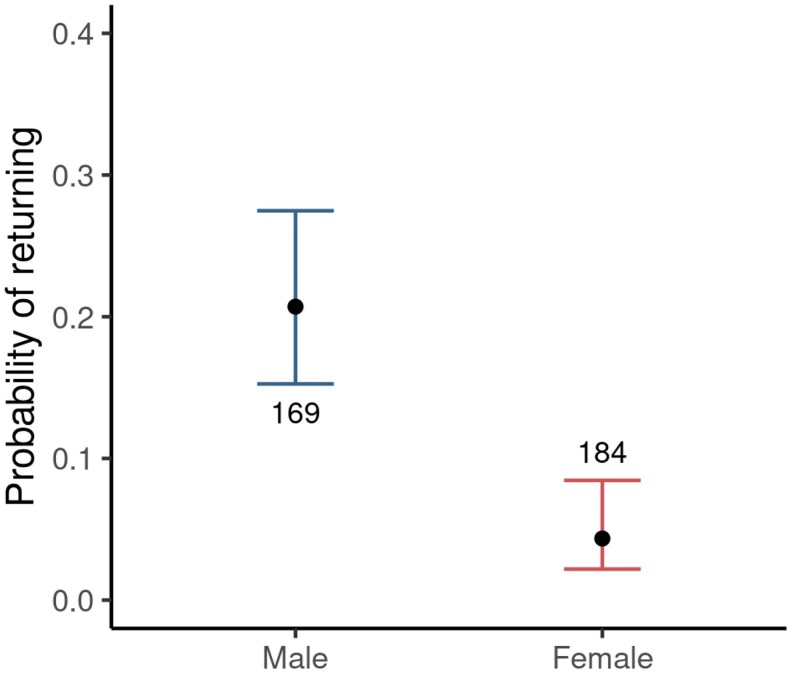
Difference between males and females in the probability of returning to the study site between breeding seasons. Error bars show model predictions and 95% confidence interval (CI), and numbers indicate the sample size.

Across the study period, 20.7% (35/169) of males and 4.3% (8/184) of females returned to the study area (Table 1, Fig. 1). The difference between males and females in the probability of returning to the study area was highly significant (Table 2). There was no difference in the probability of returning between the 2 study years, nor was there a significant sex × year interaction (Table 2).

**Table 1:** Return rates of red phalaropes to a 2.5 km^2^ study area near Utqiagvik, Alaska.

Year of banding	Sex	*n*	Returned in		Total
			2018	2019	
2017	Male	65	11 (16.9%)	10 (15.4%)^a^	15 (23.4%)
	Female	74	1 (1.4%)	1 (1.4%)	2 (2.7%)
2018	Male	104		20 (19.2%)	20 (19.6%)
	Female	110		6 (5.5%)	6 (5.6%)
Total	Male	169			35 (20.7%)
	Female	184			8 (4.3%)

“*n*” refers to the number of marked individuals.

^a^Of these individuals, 6 were also seen in 2018 and 4 were not.

**Table 2:** Sex differences in local breeding site fidelity.

Variable	Estimate	SE	*z*	*P*
*Intercept*	−3.58	0.72		
Sex (male)	2.38	0.78	3.07	0.002
Year	0.73	0.83	0.88	0.38
Sex × year	−0.96	0.92	−1.05	0.29

Results of a generalized linear model examining whether the probability that an individual returned to the study area differs between males and females. The analysis is based on 169 males and 184 females. The sex effect is relative to females and the year effect is relative to 2017.

Note that none of the 93 male and 96 female hatchlings that were banded at the study site in 2017 and 2018 returned in a subsequent year.

### Dispersal distance and mate fidelity

**Figure 2:**
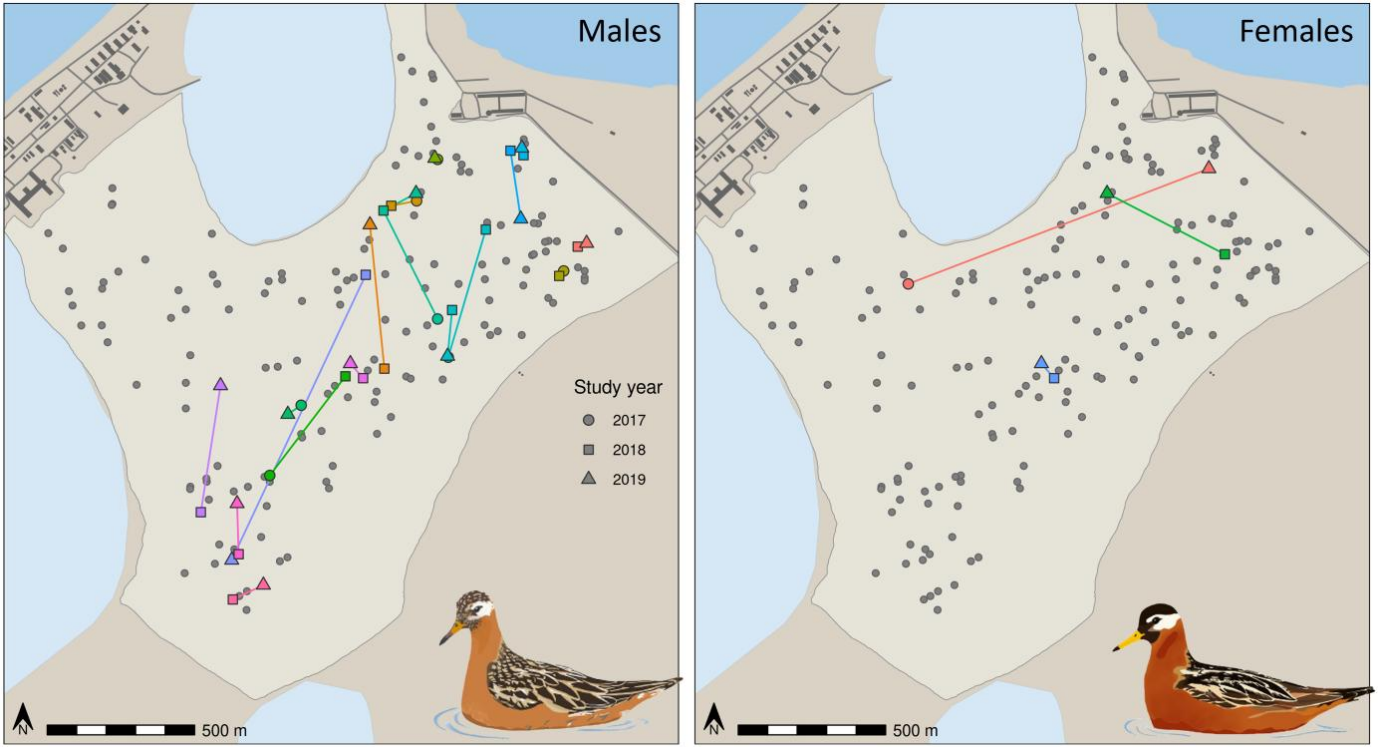
Map of the study site, showing the nest locations of individual males and females that returned between years and had a nest in both years. Each individual is represented by a different color with symbols and lines connecting the 2 nest locations. Gray dots indicate all known nest locations in the years 2017 and 2018. Tundra habitat outside the study area is indicated in darker brown, and lakes and the sea are indicated by light and dark blue, respectively. When an individual had more than 1 nest in a given year, we show the last nest of the initial year and the first nest of the return year (see Materials and Methods).

Eighteen returning males and 3 returning females had a nest in the study area in both years. The median dispersal distance was 144 m for males (range: 10 to 1,093 m) and 453 m for females (range: 67 to 1,097 m; Fig. 2). Males thus tended to have shorter dispersal distances than females, but the sample size for females is small (*n* = 3) and the difference was not statistically significant (Table S2).

In 2 cases, both members of a pair returned to the study area the following year. In both cases, their breeding attempt in the initial year had been successful (ie the male incubated until hatchlings were brought back to his nest). One of these pairs re-united and bred together again in the year of return. The distance between their nests was 67 m, and they bred again successfully in the year of their return. For the other pair, neither the male nor the female was associated with a known nest in the year of their return, so they either did not re-unite or they did but bred elsewhere.

### Predictors of breeding site fidelity

**Figure 3:**
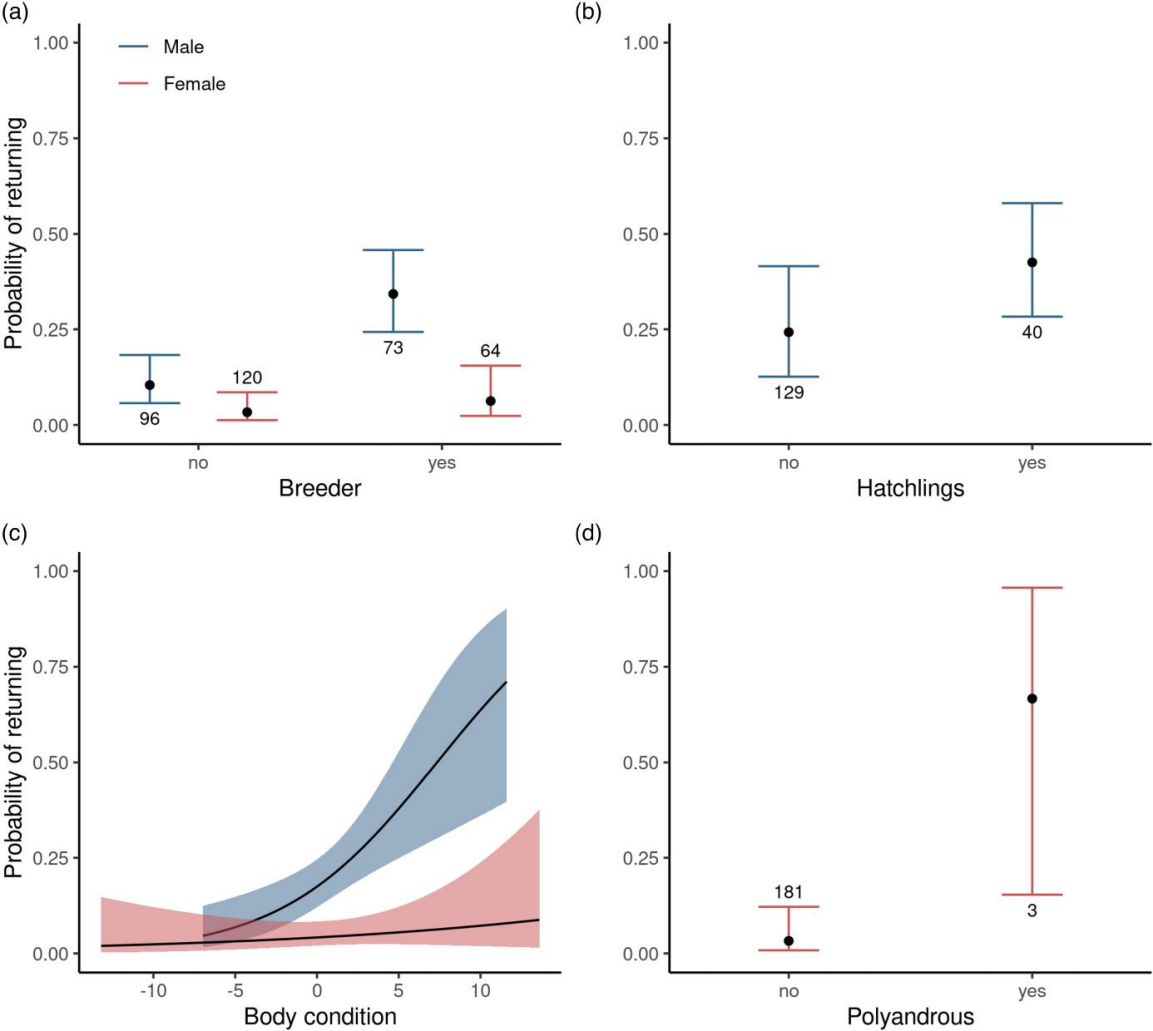
Correlates of male and female breeding site fidelity. Shown are the probability of returning to the study area between breeding seasons in relation to a) whether or not the individual bred in the study area, b) whether or not the individual incubated dummy eggs until receiving hatchlings after artificial incubation of the real eggs (males only, see Materials and Methods), c) body condition, and d) whether or not the individual was socially polyandrous (females only). Shown are model predictions (points and lines) with their 95% CI (error bars and shaded areas). Numbers indicate the sample size.

Males that bred in the study area were more likely to return (34%, 25/73) than males that did not nest in the study area (10%, 10/96) (Table 3, Fig. 3a). This effect may be confounded by tenure, because males that had a nest stayed longer at the study site than males that did not (GLM: estimate = 0.28, SE = 0.04, *z* = 6.68, *P* < 0.001). However, the effect was robust to excluding males that visited the study area only for a few days before moving on: the probability of returning was still higher for males with a nest when individuals with a local tenure of <5 d were excluded from the analyses (Fig. S1). Among males that bred in the study area, those that produced hatchlings were somewhat more likely to return (43%, 17/40) than those that did not produce hatchlings (24%, 8/33), but this difference was not statistically significant (Table 3, Fig. 3b). The probability of return was also higher for males with a higher body condition (Table 3, Fig. 3c). The probability that a male returned was unrelated to its body size and hematocrit value, and did not depend on whether the bird was tagged or not (Table 3).

**Table 3:** Predictors of male breeding site fidelity.

	Variable	Estimate	SE	*z*	*P*
All males	*Intercept*	2.61	3.87		
	Breeding (yes/no)	1.32	0.48	2.73	0.006
	Body size	−0.05	0.19	−0.26	0.79
	Body condition	0.19	0.07	2.78	0.005
	Hematocrit	−0.09	0.07	−1.23	0.22
	Tagged (yes/no)	−0.41	0.55	−0.75	0.45
Males with a nest	*Intercept*	5.61	5.70		
	Hatched (yes/no)	0.44	0.59	0.75	0.45
	Body size	−0.40	0.26	−1.52	0.13
	Body condition	0.22	0.09	2.42	0.016
	Hematocrit	−0.13	0.11	−1.19	0.23
	Tagged (yes/no)	−0.34	0.81	−0.42	0.68

Results of generalized linear models examining factors associated with the probability that an individual returned to the study area. We considered all males caught in the study area (*N* = 149) and only those that bred (incubated a clutch) in the study area (*N* = 64). Note that the sample size for these analyses is lower than the total number of males because hematocrit values (*N* = 19) and body condition (*N* = 1) were not available for all.

Females that bred in the study area were somewhat more likely to return (6.3%, 4/64) than those that did not (3.3%, 4/120), but this difference was not statistically significant (Table 4, Fig. 3a). Among females that laid a clutch in the study area, however, those that were polyandrous (produced a clutch with >1 male) were significantly more likely to return (67%, 2/3) than those that were socially monogamous (3.3%, 2/61) (Table 4, Fig. 3d). The probability of female return was not predicted by variation in body size, body condition and hematocrit value, and did not differ between tagged and untagged individuals (Table 4, Fig. 3c). Note, however, that the statistical power of these tests was low given the small number of site faithful females.

**Table 4:** Predictors of female breeding site fidelity.

	Variable				
All females	*Intercept*	−3.10	6.06		
	Breeding (yes/no)	0.64	0.78	0.82	0.41
	Body size	0.60	0.32	1.90	0.058
	Body condition	0.02	0.08	0.24	0.81
	Hematocrit	0.00	0.11	−0.01	0.99
	Tagged (yes/no)	−1.32	1.13	−1.17	0.24
Females with a nest	*Intercept*	−7.05	13.61		
	Polyandrous (yes/no)	5.06	2.06	2.46	0.014
	Body size	0.97	0.72	1.33	0.18
	Body condition	−0.17	0.20	−0.81	0.42
	Hematocrit	0.06	0.25	0.25	0.81
	Tagged (yes/no)	0.66	1.57	0.42	0.67

Results of generalized linear models examining factors associated with the probability that an individual returned to the study area. We considered all females caught in the study area (*N* = 169) and only those that bred (laid a clutch) in the study area (*N* = 60). Note that the sample size for these analyses is lower than the total number of females because hematocrit values were not available for all (*N* = 15).

### Consequences of breeding site fidelity

**Figure 4:**
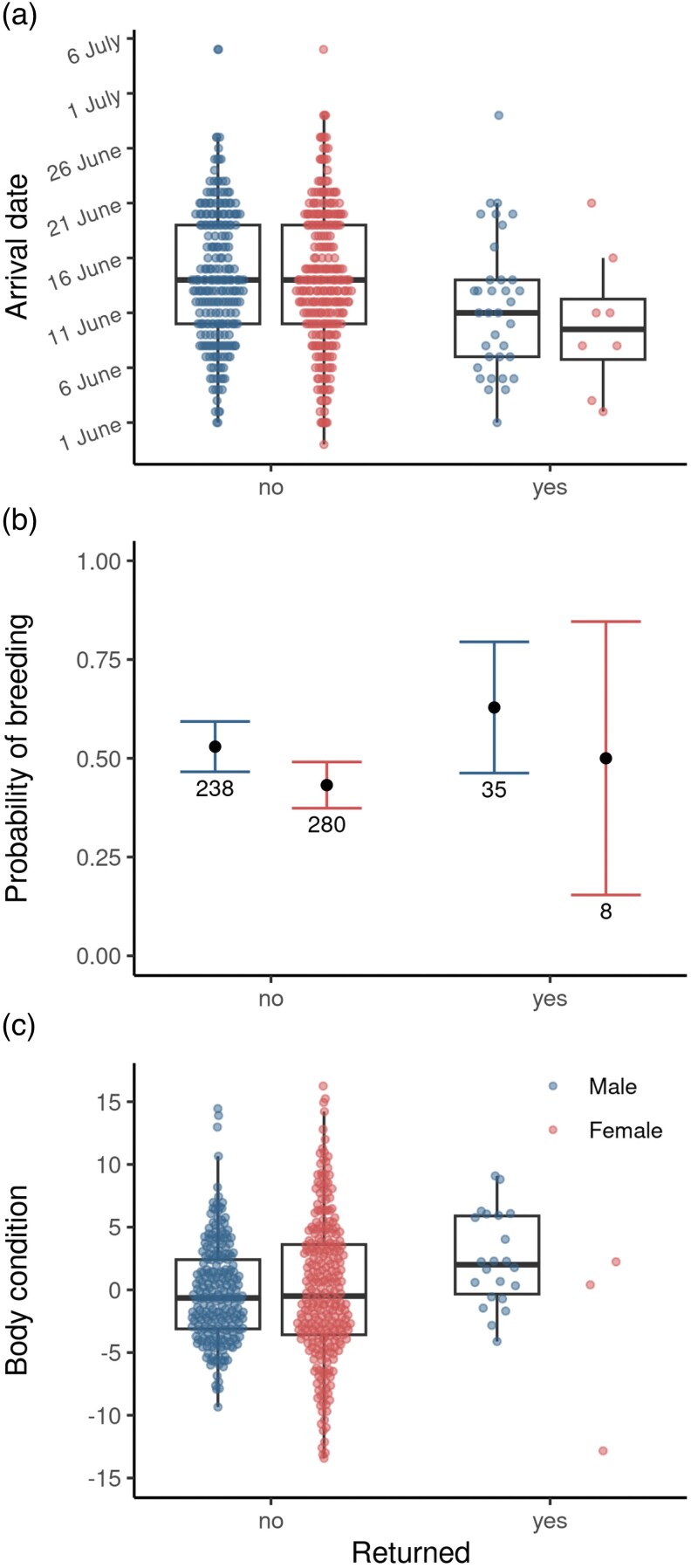
Consequences of male and female breeding site fidelity. Shown are differences between individuals that had returned (ie had been at the study site in a previous year) and those that were observed for the first time in a) arrival date, b) the probability of being a breeder, and c) body condition in the focal year (year X + 1 or X + 2). In a) model predictions and 95% CI are shown as points and error bars. In b) and c) boxplots show the median, first and third quartile and 1.5 × interquartile range. Raw data are also given as points, jittered horizontally for visibility. Numbers indicate sample sizes.

Males that had been present at the study site in previous years arrived significantly earlier (on average 2.5 d) than those that had not been observed previously in the study area (Table 5, Fig. 4a). Males that had been in the study area in previous years were also somewhat more likely to have a nest in the focal year (63%, 22/35) than males that had not been observed previously (53%, 125/235), but this difference was not statistically significant (Table 5, Fig. 4b). Among males that attended a nest, those that had been in the study area previously were equally likely to produce hatchlings (50%, 11/22) as those that had not been in the study area previously (46%, 58/125; Table 5). Males that had been present at the study site previously had a significantly higher body condition (in the focal year) than those that had not been observed in previous years (Table 5, Fig. 4c).

**Table 5:** Consequences of male breeding site fidelity.

Dependent variable	Explanatory variable	Estimate	SE	*z*/*t*	*P*
Arrival date	*Intercept*	165.210	0.396		
	Returned	−2.496	1.105	−2.260	0.025
Breeder (yes/no)	*Intercept*	0.118	0.130		
	Returned	0.408	0.373	1.094	0.27
Hatchlings (yes/no)	*Intercept*	−0.127	0.179		
	Returned	0.127	0.462	0.275	0.78
Body condition	*Intercept*	−0.239	0.252		
	Returned	2.875	0.886	3.246	0.001
	Capture date	0.064	0.044	1.470	0.14

Results of linear models examining whether arrival date, local breeding status (breeder/nonbreeder), hatching success (yes/no, for breeding males only) and body condition in the focal year differed between males that had been present at the study site in a previous year and those that had been observed for the first time. In total, 35 males had been present in a previous year, and 238 males had been observed on the study site for the first time. Among those that had a nest, the sample size was 22 and 126 males, respectively.

Females that had been present at the study site in previous years arrived on average 4.2 d earlier than those that had not been observed, but this difference was not statistically significant (Table 6, Fig. 4a). Females that had been in the study area previously were not more likely to nest locally (50%, 4/8 vs. 44%, 121/276 for first-time females; Table 6, Fig. 4b). Among females that produced a clutch in the study area, those that had been there in previous years were more likely to be socially polyandrous (25%, 1/4) than those that had not been observed (6.6%, 8/121), but the difference was not significant (Table 6). Females that had been in the study area previously also did not differ in body condition from those that had not been observed (Table 6, Fig. 4c). Note that because of the low number of site faithful females (*n* = 8), the statistical power of these tests is low. The absence of significant effects should therefore be interpreted with caution.

**Table 6:** Consequences of female breeding site fidelity.

Dependent variable	Explanatory variable	Estimate	SE	*z*/*t*	*P*
Arrival date	*Intercept*	165.164	0.379		
	Returned	−4.164	2.272	−1.833	0.068
Breeder (yes/no)	*Intercept*	−0.273	0.121		
	Returned	0.273	0.717	0.381	0.70
Polyandrous (yes/no)	*Intercept*	−2.648	0.366		
	Returned	1.549	1.211	1.279	0.20
Body condition	*Intercept*	0.035	0.330		
	Returned	−2.037	3.235	−0.630	0.53
	Capture date	0.180	0.057	3.182	0.002

Results of linear models examining whether arrival date, local breeding status (breeder/nonbreeder), mating status (monogamous/polyandrous, for breeding females only) and body condition in the focal year differed between females that had been present at the study site in a previous year and those that had been observed for the first time. In total, 8 females had been present in a previous year and 280 females had been observed on the study site for the first time. Among those that had a nest, the sample size was 4 and 121 females, respectively.

## Discussion

We color-banded 169 male and 184 female red phalaropes during 2 breeding seasons and found that the overall local return rate was significantly higher in males (20.7%) than in females (4.3%). This is consistent with the hypothesis that breeding site fidelity is male-biased in socially polyandrous species in which females do not hold a territory but defend their mate directly against other females (*female access polyandry*, Greenwood 1980; Schamel and Tracy 1991). Our findings support previous work on red phalaropes at the same study site (Schamel and Tracy 1991), which also found higher return rates in males than in females, but lacked the statistical power to detect a significant difference. Note that return rates reported in our study were overall somewhat higher than those reported in Schamel and Tracy (1991), which likely reflects methodological differences, as discussed below.

Studies on breeding site fidelity in other sex-role reversed species have produced mixed results. In the red-necked phalarope, a closely related species with a similar mating system, return rates did not differ between the sexes (Reynold and Crooke 1988; Schamel and Tracy 1991). In the spotted sandpiper *Actitis macularius*, in which females defend a territory to which they attract males (*resource defense polyandry*), return rates were also similar for males and females (Oring et al. 1983). In black coucals, *Centropus grillii*, in which females also defend a territory to which they attract males, females appear to have lower local return rates than males—contrary to Greenwood's hypothesis (Eberhart-Hertel et al. 2024). This highlights that breeding site fidelity is not simply a product of the mating system, but likely affected by multiple ecological and life-history factors (Dobson 2013; Li and Kokko 2018). However, information on both types of polyandrous mating systems is scarce and more data would be valuable.

The difference in patterns of breeding site fidelity between red-necked and red phalaropes is notable, given that these species breed sympatrically and share many life-history traits, including an almost identical mating system. In red-necked phalaropes, local return rates are much higher than in red phalaropes and do not differ between the sexes (Reynold and Crooke 1988; Schamel and Tracy 1991). The lower return rates in red phalaropes may be related to the fact that their breeding range includes more northerly latitudes where between-year variation in local breeding conditions is larger (Schamel and Tracy 1991), and in certain locations extreme conditions may prevent breeding altogether in some years (Mayfield 1978; Schamel and Tracy 1987). Such conditions likely favor flexibility in settlement decisions and, hence, may have led to lower breeding site fidelity. It is also conceivable that a higher return rate of the caring sex (males) in the red-necked phalarope—which may result from more predictable ecological conditions—may, in turn, promote site fidelity in the competing sex (females), because the distribution of mating opportunities becomes more predictable. If true, this could explain the absence of a sex difference in red-necked phalaropes.

The probability of return was associated with breeding success in both sexes. Males that bred in the study area, ie that incubated a clutch, were more likely to return than males without a nest, while locally polyandrous females were more likely to return than females that only laid a clutch for a single male at the study site. Males provide all parental care in red phalaropes, and returning males may thus benefit from familiarity with local conditions. It is less clear whether and how females would benefit from returning to the same site. Higher breeding site fidelity for successful individuals has also been reported for males in polygynous species in which females provide all parental care (Höglund and Robertson 1990; Widemo 1997; Vervoort and Kempenaers 2019; Santema et al. 2025). However, in these species, males defend a display territory or a position on a lek to which they attract females, and returning males may thus benefit from a residency advantage. Female red phalaropes do not defend a territory or other resources, so benefits due to prior residency do not play a role. It is possible that the higher return rate of successful females simply results from a link between within-season and between-season site tenacity (ie strength of attachment to a site). Females that had multiple clutches within the study site by definition showed high within-season site tenacity. If these females generally show a higher degree of site tenacity, ie both within and between years, this could explain the higher return rate of females with multiple clutches. We emphasize that our measure of female mating success only considers the local situation: females that had only a single mate at the study site were not necessarily less successful overall, because they may have mated elsewhere.

Returning males and females, ie those that had been at the study site in a previous year, arrived earlier than individuals that were recorded at the study site for the first time, although this effect was statistically significant only for males. Early arrival at the breeding site is associated with higher breeding performance in many species (Morbey and Ydenberg 2001), but we did not find evidence for this effect in red phalaropes (relationship between arrival date and probability of breeding locally; GLM; males: estimate = −0.03, SE = 0.02, *z* = −1.21, *P* = 0.23; females: estimate = 0.01, SE = 0.02, *z* = 0.80, *P* = 0.43). The lack of a relationship between arrival and probability of breeding might be a result of our definition of arrival date (see Materials and Methods), and we cannot exclude that individuals that arrived earlier to the general area (beyond the study site) were more likely to breed locally. We also did not find evidence that returning individuals did better: they were not more likely to breed, and, among those that bred, were not more likely to acquire multiple mates (females) or to successfully incubate until receiving hatchlings (males). However, with low local return rates, the sample sizes were small (*N* = 35 males, 8 females), such that our study may have lacked the statistical power to detect an effect. The observed effects were positive for all breeding performance parameters (Tables 5 and 6) suggesting that, if anything, returning individuals performed better than individuals that were new to the area.

Despite the low return rate in both sexes, there were 2 cases where both members of a breeding pair returned to the study area the following year. Remarkably, the male and female of 1 pair re-united to breed again in the year of their return and in both years their breeding attempts were successful. Reynolds and Cooke (1988) found that of 6 red-necked phalarope pairs of which both individuals returned to the same site between years, 2 re-united to form a pair again in the second year. The benefits of mate reunion are thought to be an important driver for breeding site fidelity in other shorebird species (Kwon et al. 2022), but related to coordination of incubation duties. Although anecdotal, these observations suggest that individuals may preferentially mate with a previous partner when they have the opportunity, even in uniparental species with low site fidelity where the likelihood of encountering a previous partner is low. Whether and how individuals benefit from remating with the same partner in such systems remains unclear.

Inter-annual variation in local breeding conditions may be an important factor underlying settlement decisions in red phalaropes, and the conditions during the years in which our study took place may thus have affected return rates. The first 2 yr of our study (2017 and 2018) were characterized by late snowmelt, low phalarope breeding densities, and low levels of social polyandry, whereas the last year (2019) was characterized by early snowmelt, high phalarope density, and a high level of social polyandry (Krietsch et al. 2022). Males banded in 2017 and 2018 had similar return rates, but females banded in 2018 were somewhat more likely to return the following year (5.5%, 6/110) than those banded in 2017 (1.4%, 1/74). Although this difference was not statistically significant (GLM; estimate = 1.44, SE = 1.09, *z* = 1.32, *P* = 0.19), it suggests that return rates of females may have been higher in the year with early snowmelt and a high breeding density (ie 2019). Given the high rate of social polyandry in 2019, the proportion of females that returned in the following year may also have been higher, but unfortunately we were unable to monitor their return due to travel restrictions related to the COVID-19 pandemic.

Our observed levels of breeding site fidelity may also have been affected by the fact that our study site is part of an expansive area of continuous breeding habitat. Individuals may thus have returned to the general area, but settled outside the 2.5 km^2^ study site. Indeed, some individuals that had been equipped with GPS tags dispersed outside the study area within the same season (our unpublished data), and individuals that returned also dispersed within the study area (Fig. 2). Notably, however, several males returned to nest in almost the exact same location as where they nested the previous year (Fig. 2). The fact that our study site is part of a much larger area of suitable breeding habitat may explain the difference in breeding site fidelity with a small isolated population of red phalaropes in Iceland, where all of 4 color-banded males returned to the study site the following year (Whitfield 1995). These observations suggest that site fidelity can be flexible within species and is linked to the extent of suitable breeding habitat, which influences opportunities for breeding dispersal.

Our local return rate estimates likely provide a lower limit of the true return rate of red phalaropes for several reasons. First, some individuals may not have survived the nonbreeding season, such that the return rate of individuals that survived would be underestimated (Cormack 1964; Martin et al. 1995). Because shorebirds generally have relatively high annual survival rates (Mendez et al. 2018), the contribution of mortality to the low return rate is likely small. Second, some returning individuals that stayed only briefly at the study site may have remained undetected. Of the birds that we caught and color-banded, 21% of males and 37% of females were never seen again. Of the birds that returned in a following year, too, 17% of males (6/35) and 25% of females (2/8) were observed only once. It is thus plausible that some individuals that returned to the study area—especially those that stayed only briefly—were not observed at all, despite our efforts to systematically survey the entire area. Third, as we noted above, it is possible that some individuals returned to the general area, but settled just outside the study site (Martin et al. 1995). Local dispersal may have a stronger effect on estimates of female return rates, if breeding dispersal is larger for females than for males. Although the median distance moved by females within the study area was larger for females (453 m) than for males (144 m), the sample size for females is small and the difference is not statistically significant. Three males that had returned (ie were observed on the study site) were subsequently found on a nest outside the study area (nearest distance to the study area: 560, 617, and 859 m). It is thus possible that other returning individuals bred outside the study area and were never seen. The fact that returning birds still dispersed locally may explain why the return rates we observed are higher than those reported by Schamel and Tracy (1991) for the same population. Given that our study area was substantially larger (2.5 km^2^ compared with 1 km^2^), individuals that dispersed locally would have been more likely to remain within its boundaries. These observations highlight that study area size is an important factor to consider when comparing return rates between species, populations, or different studies of the same population (see also McCaffery and Ruthrauff 2004). Finally, our estimates of local return rates may also have been affected by predation rates. Although the difference was not statistically significant, males whose clutch was predated were less likely to return. If predation on natural clutches would have been higher (compared with those with dummy eggs), male return rates might have been lower. Note, however, that the risk of predation probably increases with the number of nest visits. Hence, local return rates may be higher under natural circumstances (without human presence).

In summary, we show that in the red phalarope, a sex-role reversed species in which females directly compete among each other for access to mates (*female access polyandry*), the likelihood of returning to a former breeding site is low for both sexes, but substantially higher for males. These findings are consistent with the prediction that site fidelity should be male-biased in polyandrous species in which females defend individual mates rather than resources. The probability of return increased with local breeding success for both sexes: nesting males were more likely to return than nonnesting males, and, among nesting females, those that were socially polyandrous were more likely to return than those that only bred with 1 male locally. These results suggest that breeding site fidelity is associated with local success, even in a nonterritorial, polyandrous species that breeds in a highly unpredictable environment.

## Supplementary Material

arag090_Supplementary_Data

## Data Availability

Analyses reported in this article can be reproduced using the data and code provided by Santema et al. (2026).
