# Supplementary material for: Factors associated with breeding site fidelity in the sex-role reversed red phalarope
Source: Behav Ecol. 2026 Jul 28;37(5):arag090. doi: 10.1093/beheco/arag090 (PMC13455084; doi:10.1093/beheco/arag090)

Supplementary Materials to:

Factors associated with breeding site fidelity in the sex-role reversed red phalarope

Santema, P., Krietsch, J., Kempenaers, B.

Behavioral Ecology, 2026

Contents

Table S1.. 2

Table S2.. 3

Figure S1.. 4

Table S1. Overview of the number of observers in the field during each day of the study period.

date	2017	2018	2019
01-May	0	0	0
02-May	0	0	0
03-May	0	0	0
04-May	0	0	0
05-May	0	0	0
06-May	0	0	0
07-May	0	0	0
08-May	0	0	0
09-May	0	0	0
10-May	0	0	0
11-May	0	0	0
12-May	0	0	0
13-May	0	0	0
14-May	0	0	0
15-May	0	0	0
16-May	0	0	0
17-May	0	3	0
18-May	3	3	0
19-May	3	3	0
20-May	3	3	0
21-May	3	3	0
22-May	3	3	3
23-May	3	3	3
24-May	3	3	3
25-May	3	3	3
26-May	3	3	3
27-May	3	5	5
28-May	9	5	5
29-May	9	5	5
30-May	9	5	5
31-May	9	5	5

date	2017	2018	2019
01-Jun	9	5	5
02-Jun	9	5	5
03-Jun	9	5	7
04-Jun	9	10	9
05-Jun	9	10	9
06-Jun	9	10	9
07-Jun	9	10	9
08-Jun	9	10	9
09-Jun	9	10	9
10-Jun	9	10	9
11-Jun	9	10	9
12-Jun	9	10	10
13-Jun	9	10	10
14-Jun	9	10	10
15-Jun	9	10	10
16-Jun	9	10	10
17-Jun	9	10	10
18-Jun	9	10	10
19-Jun	9	10	10
20-Jun	9	10	10
21-Jun	9	10	10
22-Jun	9	10	10
23-Jun	9	10	10
24-Jun	9	10	10
25-Jun	9	8	10
26-Jun	9	8	10
27-Jun	9	8	10
28-Jun	8	7	10
29-Jun	8	7	8
30-Jun	8	7	7

date	2017	2018	2019
01-Jul	6	7	7
02-Jul	5	4	7
03-Jul	5	4	7
04-Jul	5	4	7
05-Jul	4	4	7
06-Jul	4	4	7
07-Jul	4	4	7
08-Jul	4	4	7
09-Jul	4	4	7
10-Jul	4	4	7
11-Jul	4	4	7
12-Jul	3	3	7
13-Jul	3	3	7
14-Jul	3	3	7
15-Jul	3	3	6
16-Jul	3	3	6
17-Jul	3	3	6
18-Jul	3	3	3
19-Jul	3	3	3
20-Jul	3	3	3
21-Jul	3	3	3
22-Jul	0	0	3
23-Jul	0	0	3
24-Jul	0	0	3
25-Jul	0	0	3
26-Jul	0	0	3
27-Jul	0	0	3
28-Jul	0	0	3
29-Jul	0	0	1
30-Jul	0	0	1
31-Jul	0	0	1

Table S2. Sex differences in breeding dispersal distance. Results of a generalised linear model examining the effect of sex on variation in dispersal distance (in meters) within the study site. The analysis is based on only 19 males and 3 females that bred in two years. The sex effect is relative to female.

Variable	Estimate	SE	z	p
Intercept	538.90	243.70		
Sex (male)	-218.60	262.20	-0.83	0.41

Figure S1. Results of a sensitivity analysis showing how excluding males with a short tenure at the study site affected the relationship between breeding in a given year (yes/no) and returning to the study area in the next year (yes/no). Shown are effect size estimates (a) and z-values (b) for models where males with tenures between one and up to ten days were excluded. Z-values >1.96 (indicated with dotted horizontal line) are statistically significant (p <0.05). Numbers in (b) indicate sample sizes for each test. The effect of breeding on return in the next year was no longer significant when males that stayed for up to five days were excluded, which may partly reflect the reduced sample size.

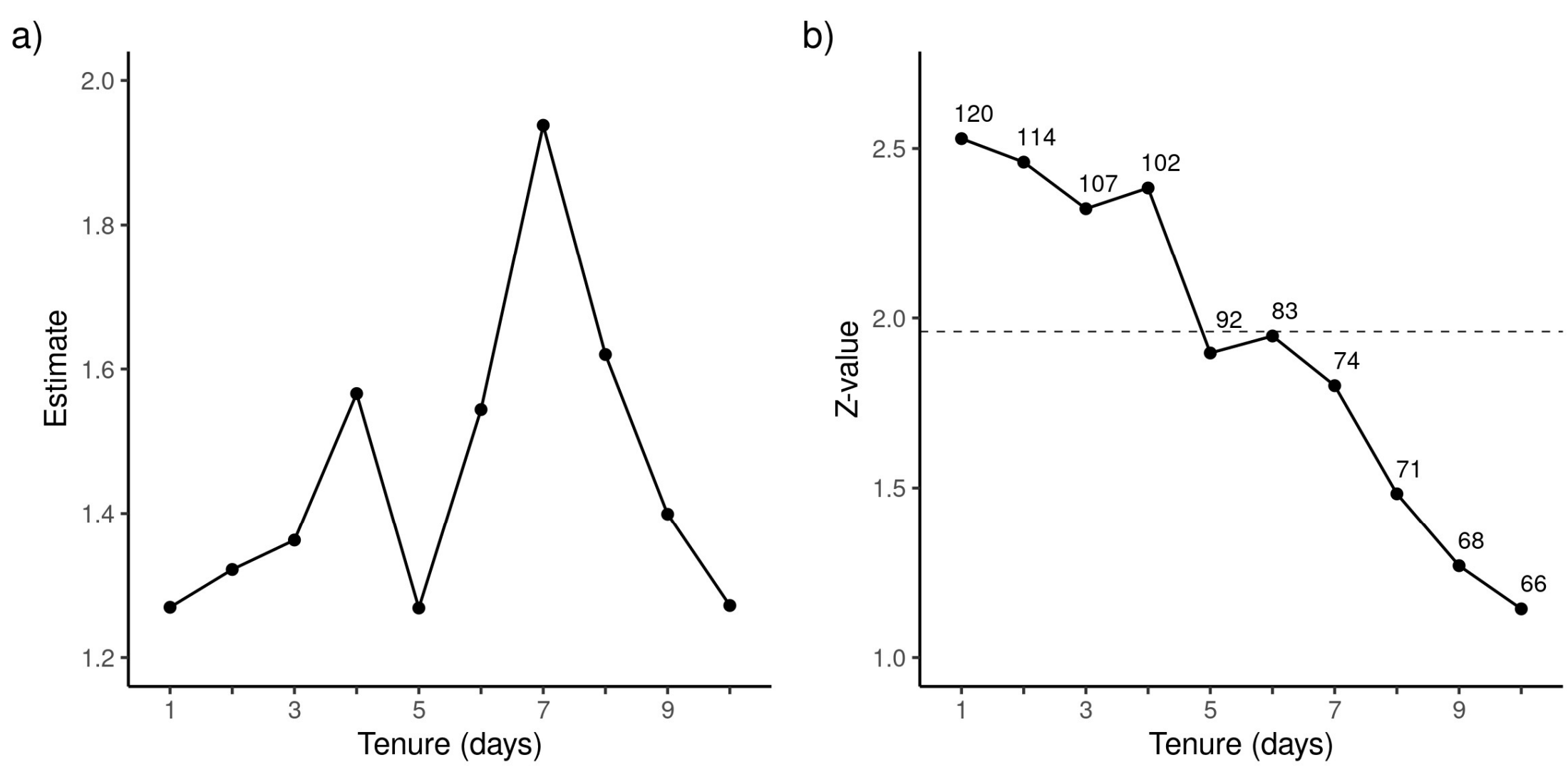

Supplement: Supplementary file 1 [file arag090_supplementary_data.pdf]
